# Accurate and affordable cobot calibration without external measurement devices

**DOI:** 10.1038/s44172-026-00633-4

**Published:** 2026-04-06

**Authors:** Giovanni Franzese, Max Spahn, Jens Kober, Javier Alonso-Mora, Cosimo Della Santina

**Affiliations:** 1 Technology Innovation Institute, Abu Dhabi, United Arab Emirates; 2 ABB Robotics, Mannheim, Germany; 3 University of Stuttgart, Stuttgart, Germany; 4 Delft University of Technology, Delft, The Netherlands

**Keywords:** Mechanical engineering, Computational science

## Abstract

To reliably perform everyday tasks, collaborative robots must be accurate, not merely repeatable. Unfortunately, precise kinematic calibration often relies on tools that are more expensive than the robots themselves. We address this limitation by proposing a low-cost and effective calibration method aimed at democratizing cobot calibration. Our minimalist approach uses a single 3D-printable two-socket spherical-joint tool to kinematically constrain the robot end effector during data collection. An optimization routine updates the nominal kinematic model to ensure consistent socket predictions while preserving their mean distance. We validate the method on Franka, KUKA, and Kinova cobots, consistently reducing mean absolute errors, for example, from approximately 10 mm to 0.2 mm on Franka robots. To demonstrate practical impact, we further evaluate the calibrated model on a Franka robot in a peg-in-the-hole task with 0.4 mm tolerance and in a repeated drawing task using Cartesian control and learning from demonstration. Both tasks fail without calibration and consistently succeed with the calibrated model. The proposed method enables affordable and practical cobot calibration, providing a foundation for accurate manipulation tasks.

## Abstract (web summary)

Giovanni Franzese and colleagues introduce a low-cost calibration method using a two-socket and spherical-joint tool. Validated on various robots, the method reduces errors by up to two orders of magnitude, enabling the successful execution of high-precision manipulation tasks.

## Introduction

To face the challenge of modern manipulation tasks in unstructured environments, where robots need to adapt their behavior in the Cartesian space, we require robots that are not only repeatable but also accurate. However, due to manufacturing and assembly tolerances, wear, and bending, errors occur over the entirety of the kinematic chain.

Kinematic errors are especially harmful in serial manipulators, where the parameter deviations propagate through the kinematic chain, resulting in wrong end-effector prediction. This is a known and well-studied problem^1^, and many calibration routines for industrial robots have been proposed, including Coordinate-Measuring Machines (CMMs)^2^ for high precision but low portability, laser trackers^3,4^ for real-time tracking at high cost, and Motion Capture^5^ or standard cameras^6^ for concept validation but limited precision. However, these tools are not portable, require an expert to set up, and are expensive. For this reason, when working with affordable collaborative robots, the calibration of the kinematics is not considered worth the investment.

**Fig. 1:**
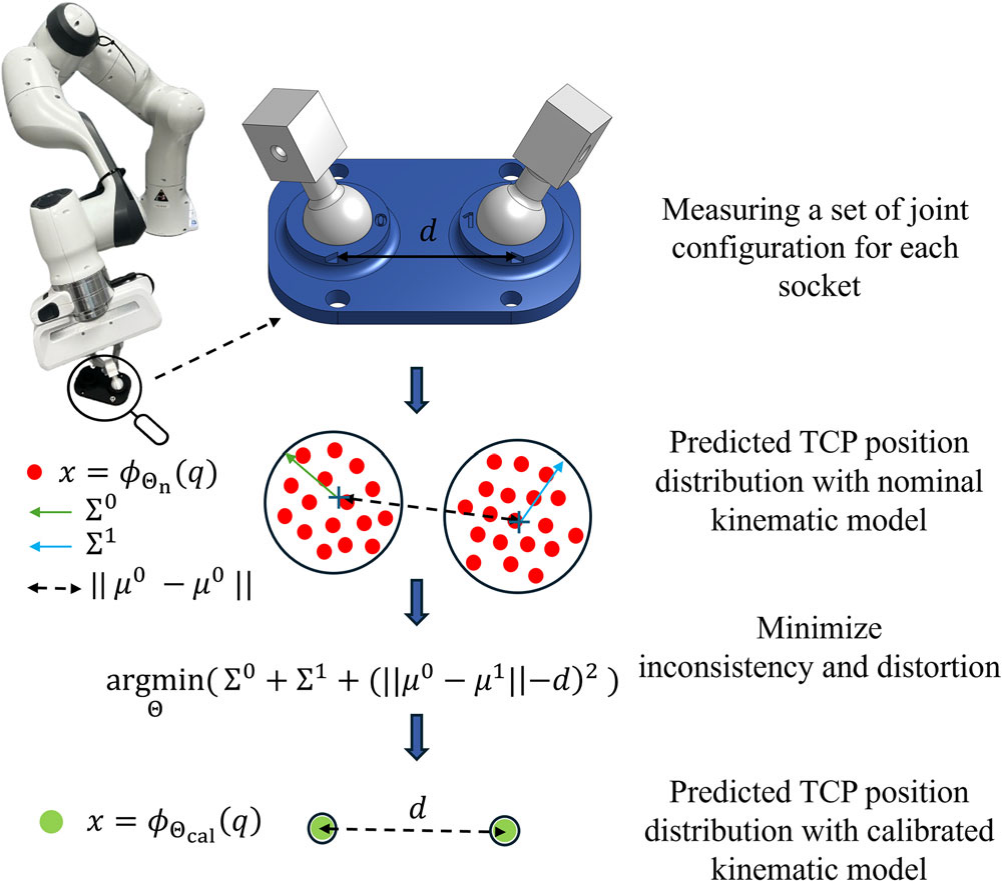
MUKCa tool and the proposed calibration routine. The optimization minimizes the inconsistency and distortion of the predicted ball position attached to the end-effector for each of the two sockets.

This article’s contribution is the Minimalist and User-friendly Kinematics Calibration (MUKCa) tool, as the combination of an affordable calibration device and an optimization algorithm for kinematic parameter identification, as illustrated in Fig. 1. The proposed minimalist tool is designed for accessibility and affordability, and it is 3D printable. Moreover, it is composed solely of a sphere (or ball) with a two-socket base, without relying on external measurement systems.

After printing the tools and placing the sockets in front of the robot and the sphere attached to the end effector, the data collection can begin immediately. The user is asked to kinesthetically place the ball in each of the sockets and record different joint configurations that satisfy the requirement. The optimization procedure is the first of its kind in the field of robot calibration, simultaneously minimizing Cartesian inconsistency and volumetric distortion, and it only relies on the manufacturing distance between the sockets. The resulting models enable redundant robots to reliably perform high-precision tasks across different joint configurations for a given Cartesian task and to generalize these tasks to novel Cartesian configurations.

In the literature, a similar setup is the three-metal sphere artifacts^7–9^. The main idea is that the metal spheres are manufactured to be perfectly spherical and are placed at a known precise distance from each other. The spherical constraints of the metallic surface and the sphere’s radius are used to update the kinematic model. However, the points on the surface of each sphere are recorded using a precision probe^7^ to increase accuracy, making the setup unaffordable for every pocket. Additionally, the data collection often requires more than 30 minutes. To eliminate the use of the precision probe, three digital indicators, fixed orthogonally to each other, have been proposed^8^. Rather than reconstructing the surface of the metal ball, the digital indicator is used to automatically align the robot TCP with the center of each of the three balls on the plate. Despite the portability of the tool, precision sensors are still required to perform the automatic alignment, which keeps the cost prohibitive. To avoid the usage of expensive measuring devices, an alternative is to use a kinematic coupling device, i.e., a magnetic socket attached to the end effector that perfectly fits each of the three spheres^9^. The kinematic calibration is then performed by solving an optimization problem, where the fitting cost measures the error in the estimated distances between the sphere and the manufacturing distance. Since the achieved accuracy relies directly on the tool’s accuracy, its calibration faces similar limitations as the previously mentioned methods, particularly in terms of cost and portability.

Recently, a novel approach highlighted spherical joints as a cost-effective alternative to the three-sphere system^10^. The proposed method involves measuring different configurations with an end-effector ball in various sockets placed within the workspace. However, since the sockets are hand-positioned, it is not possible to know the accurate distance between them. The proposed fitting function is the minimization of the discrepancy between the predicted forward kinematic position of each socket and the average value of the nominal model. However, the use of the nominal model as ground truth is a strong limitation that retains volumetric miscalibration; hence, our proposed method relaxes this assumption.

In summary, our proposed method combines the cost-effectiveness of spherical joints while:

- eliminating the limitation of relying on the average nominal model^10^,
- avoiding the need for an expensive CMM to calibrate the distance between the sockets^8^ or using an external measure to define the calibration tool frame, and
- removing the requirement for a costly auto-centering device for the spherical joint in the socket^9^.

Instead, we propose a low-cost alternative where the user kinesthetically positions the ball within the socket for different joint configurations. This compromise provides the best balance between affordability, accuracy, and practicality.

## Methods

We first describe the forward kinematics formulation and its parametrization. Next, we outline our data collection process and key assumptions, followed by a detailed description of the optimization procedure and the related assumptions.

### Kinematics

A kinematic chain is composed of *m* links and *n* joints, where *m*≥*n*. A transformation between two frames *a* and *b* on the kinematic chain is described by a homogeneous transformation matrix ${{{\boldsymbol{T}}}}_{a}^{b}$. The frame 0 represents the robot’s base frame, and some frames are referred to by a descriptive name, such as *sphere* or *ball*. We refer to ${{{\boldsymbol{X}}}}_{a}^{b}$ as the translational part of the transformation, in other words, the last column of the matrix excluding the 4th row. There are generally two types of joints: (a) fixed joints, where the corresponding transformation matrix is purely dependent on the geometry, and (b) actuated joints, where the transformation matrix is additionally dependent on the joint value.

The transformation matrix for a fixed joint between frame *j*−1 and *j* can be obtained from the Roll-Pitch-Yaw angles [*α*, *β*, *γ*]_*j*−1_ and the displacement vector ${{{\boldsymbol{p}}}}_{j-1}={[{p}_{x},{p}_{y},{p}_{z}]}_{j-1}$ as:

1 $${{{\bf{T}}}}_{j-1}^{j}=\left[\begin{array}{cc}{{\boldsymbol{R}}}({[\alpha ,\beta ,\gamma ]}_{j-1}) & {{{\boldsymbol{p}}}}_{j-1}\\ {{{\boldsymbol{0}}}}^{\top } & 1\end{array}\right].$$

For a revolute joint defined by an axis of rotation ***a***_*j*_ and the angle *q*_*j*_, the transformation is multiplied from the right with an additional rotation, namely:

2 $${{{\boldsymbol{T}}}}_{j-1}^{j}=\left[\begin{array}{cc}{{\boldsymbol{R}}}({[\alpha ,\beta ,\gamma ]}_{j-1})\cdot {{\boldsymbol{R}}}({{{\boldsymbol{a}}}}_{j},{q}_{j}) & {{{\boldsymbol{p}}}}_{j-1}\\ {{{\boldsymbol{0}}}}^{\top } & 1\end{array}\right],$$

where

3 $${{\boldsymbol{R}}}({{{\boldsymbol{a}}}}_{j},{q}_{j})={{\boldsymbol{I}}}+\sin {q}_{j}\,{{\boldsymbol{K}}}+(1-\cos {q}_{j})\,{{{\boldsymbol{K}}}}^{2},$$

and the skew-matrix ***K*** is given by

4 $${{\boldsymbol{K}}}=\left[\begin{array}{ccc}0 & -{a}_{j,2} & {a}_{j,1}\\ {a}_{j,2} & 0 & -{a}_{j,0}\\ -{a}_{j,1} & {a}_{j,0} & 0\end{array}\right],$$

while the vector ***a***_*j*_ is the unit axis of rotation for the *j*th joint. For prismatic joints, similar formulas can be found in the literature^11^. The last frame on the kinematic chain, frame *m* is then obtained by the product of transformations

5 $${{{\boldsymbol{T}}}}_{0}^{m}={{{\boldsymbol{T}}}}_{0}^{1}{{{\boldsymbol{T}}}}_{1}^{2}\cdot \cdot \cdot {{{\boldsymbol{T}}}}_{m-1}^{m}.$$

In our approach, we use kinematic models that are parameterized by the Euler angles [*α*, *β*, *γ*]_*j*_ and the displacements ***p***_*j*_ = [*x*, *y*, *z*]_*j*_ ∀ *j* = 0, . . . , *m*. This is a deliberate choice over other parametrizations, such as Denavit-Hartenberg parameters^10^, the Product of Exponentials^3^, Vector Inner Product Error Model^12^, Linear Finite Screw Deviation Model^13^ or using Lie Theory^14^, because most robot models are available in the Universal Robot Description Format (URDF). To favor simplicity of the method over minimalistic parametrization, we avoid the conversion between the representations^15^. Finally, we concatenate all parameters into one parameter vector ${{\boldsymbol{\Theta }}}\in {{{\mathcal{R}}}}^{6m}$, such that we can write the parameterized forward kinematics as a function of the parameters **Θ** and the joint angles ***q***, i.e., ${{{\boldsymbol{T}}}}_{0}^{m}({{\boldsymbol{\Theta }}},{{\boldsymbol{q}}})$.

### Data collection and assumptions

**Fig. 2:**
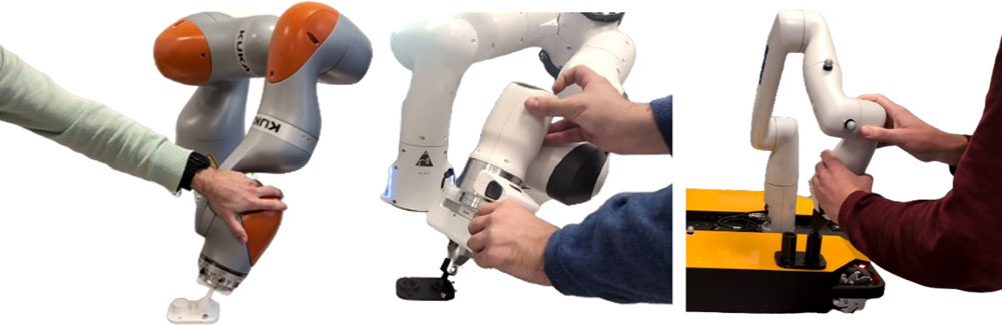
Data collection routine. The user records a set of joint configurations for each of the two sockets of the MUKCa tool. From left to right: Kuka iiwa 14, Franka Robot, and Kinova Gen3Lite.

We record two sets of configurations, one with the sphere in the socket “zero” and one in the socket “one”. Figure 2 depicts the users who kinesthetically move the robot in the nullspace and record the configuration that results in the sphere being positioned in one of the two sockets. This brings the

#### Assumption 1

The robot’s motors are back-drivable.

This is a key component of the user-friendliness of the proposed methodology that does not require designing an ad hoc controller or planning strategy to satisfy that constraint. We identify the two datasets with the corresponding apex, i.e. ${{{\boldsymbol{q}}}}^{k}\in {{\mathbb{R}}}^{1\times m}$ where *m* is the number of joints in the kinematic chain and *k* is the index of the socket where the kinematic chain terminates. Given a set of kinematic parameters, the predicted ball center point (BCP) for each *i*th joint configuration in each socket, respectively, is

6 $${{{\boldsymbol{X}}}}_{i}^{0}={\Phi }_{{{\boldsymbol{\Theta }}}}\left({{{\boldsymbol{q}}}}_{i}^{0}\right)\,{{\rm{and}}}\,\,{{{\boldsymbol{X}}}}_{i}^{1}={\Phi }_{{{\boldsymbol{\Theta }}}}\left({{{\boldsymbol{q}}}}_{i}^{1}\right)$$

where *Φ*_**Θ**_ is the parameterized forward kinematics of the BCP and ${{{\boldsymbol{X}}}}_{i}^{0,1}$ are the predicted Cartesian position of the sockets. However, the necessity to record a set of configurations larger than one that have the BCP in the same sockets implies the

#### Assumption 2

The robot is kinematically redundant.

This means that the robot can reach the same end-effector position with different joint configurations. The violation of this assumption would make the recording of a set of measurements for each socket impossible.

### Parameter calibration

Given a vector of parameters **Θ** and *N*^0^ and *N*^1^ measured joint configurations for the socket 0 and 1, the mean square error (MSE) with respect to the ground truth Cartesian position $\widehat{{{\boldsymbol{X}}}}$ is

7 $${J}_{{{\rm{mse}}}}=\frac{1}{N}\mathop{\sum }\limits_{i=1}^{N}{\parallel {\Phi }_{{{\boldsymbol{\Theta }}}}({{{\boldsymbol{q}}}}_{i})-{\widehat{{{\boldsymbol{X}}}}}_{i}\parallel }_{2}^{2}$$

where *N* = *N*^0^ + *N*^1^. However, we know that the BCP is located in only two positions; hence, we can rewrite the MSE as

8 $$\frac{1}{N}\left(\mathop{\sum }\limits_{i=1}^{{N}^{0}}{\left\Vert {\Phi }_{{{\boldsymbol{\Theta }}}}({{{\boldsymbol{q}}}}_{i}^{0})-{\widehat{{{\boldsymbol{X}}}}}^{0}\right\Vert }_{2}^{2}+\mathop{\sum }\limits_{i=1}^{{N}^{1}}{\left\Vert {\Phi }_{{{\boldsymbol{\Theta }}}}({{{\boldsymbol{q}}}}_{i}^{1})-{\widehat{{{\boldsymbol{X}}}}}^{1}\right\Vert }_{2}^{2}\right)$$

that can be rewritten as the weighted sum of the mean squared error with respect to each of the holes, i.e.,

9 $${J}_{{{\rm{mse}}}}=\frac{1}{N}\left({N}^{0}{J}_{{{\rm{mse}}}}^{0}+{N}^{1}{J}_{{{\rm{mse}}}}^{1}\right).$$

By using the variance-bias decomposition^16^ of each of the two mean squared error costs, we obtain

10 $${J}_{{{\rm{m}}}{{\rm{s}}}{{\rm{e}}}}^{0} = \underbrace{{{\rm{t}}}{{\rm{r}}}({{{\boldsymbol{\Sigma }}}}^{0})}_{{{\rm{v}}}{{\rm{a}}}{{\rm{r}}}{{\rm{i}}}{{\rm{a}}}{{\rm{n}}}{{\rm{c}}}{{\rm{e}}}} + \underbrace{||{{{\boldsymbol{\mu }}}}^{0}-{\hat{{{\boldsymbol{X}}}}}^{0}|{|}_{2}^{2}}_{{{\rm{b}}}{{\rm{i}}}{{\rm{a}}}{{\rm{s}}}}$$

where tr() is trace operator. The trace of the variance matrix measures the expected squared radius of the cloud of predictions. The mean vectors ${{\boldsymbol{\mu }}}\in {{\mathbb{R}}}^{3}$ and the variance matrices ${{\boldsymbol{\Sigma }}}\in {{\mathbb{R}}}^{3\times 3}$ are a function of the optimization variable **Θ** and are defined as

11 $${{{\boldsymbol{\mu }}}}^{0,1}=\frac{1}{{N}^{0,1}}\mathop{\sum }\limits_{i=1}^{{N}^{0,1}}{\Phi }_{{{\boldsymbol{\Theta }}}}\left({{{\boldsymbol{q}}}}_{i}^{0,1}\right)$$

and

12 $${{{\boldsymbol{\Sigma }}}}^{0,1}={\mathbb{E}}\left[\left({\Phi }_{{{\boldsymbol{\Theta }}}}({{{\boldsymbol{q}}}}^{0,1})-{{{\boldsymbol{\mu }}}}^{0,1}\right){\left({\Phi }_{{{\boldsymbol{\Theta }}}}({{{\boldsymbol{q}}}}^{0,1})-{{{\boldsymbol{\mu }}}}^{0,1}\right)}^{\top }\right].$$

However, the calculation of the bias requires the measurement of the absolute position of the socket, i.e., ${\widehat{{{\boldsymbol{X}}}}}^{0,1}$, which is not readily available without relying on an expensive external measurement device.

Nevertheless, we do have an alternative ground truth measure, which is the distance between the two sockets, *d*. To ensure the distance preservation of the final model, we can minimize the bias in the estimation of the distance between the sockets, i.e.,

13 $${J}_{{{\rm{bias}}}}={({\parallel {{{\boldsymbol{\mu }}}}^{1}-{{{\boldsymbol{\mu }}}}^{0}\parallel }_{2}-d)}^{2}.$$

Hence, the proposed cost function becomes

14 $$J = 	 \, \overbrace{({N}^{0}{{\rm{t}}}{{\rm{r}}}({{{\boldsymbol{\Sigma }}}}^{0})+{N}^{(1)}{{\rm{t}}}{{\rm{r}}}({{{\boldsymbol{\Sigma }}}}^{1}))/({N}^{0}+{N}^{1})}^{{{\rm{i}}}{{\rm{n}}}{{\rm{c}}}{{\rm{o}}}{{\rm{n}}}{{\rm{s}}}{{\rm{i}}}{{\rm{s}}}{{\rm{t}}}{{\rm{e}}}{{\rm{n}}}{{\rm{c}}}{{\rm{y}}}\,{\sigma }^{2}} \\ 	 + \underbrace{(\Vert {{{\boldsymbol{\mu }}}}^{1}-{{{\boldsymbol{\mu }}}}^{0}{\Vert }_{2}-d)^{2}}_{{{\rm{d}}}{{\rm{i}}}{{\rm{s}}}{{\rm{t}}}{{\rm{o}}}{{\rm{r}}}{{\rm{t}}}{{\rm{i}}}{{\rm{o}}}{{\rm{n}}}\,{\epsilon }^{2}}+\lambda \underbrace{\Vert {{\boldsymbol{\Theta }}}-{{{\boldsymbol{\Theta }}}}_{{\mbox{n}}}{\Vert }^{2}}_{{{\rm{r}}}{{\rm{e}}}{{\rm{g}}}{{\rm{u}}}{{\rm{l}}}{{\rm{a}}}{{\rm{r}}}{{\rm{i}}}{{\rm{z}}}{{\rm{a}}}{{\rm{t}}}{{\rm{i}}}{{\rm{o}}}{{\rm{n}}}}.$$

To summarize, we minimize three terms:

1. The inconsistency (or variance), denoted *σ*^2^, tries to minimize the predicted spread of the ball center point that must consistently result in one socket or another;
2. the distortion (or bias), denoted *ϵ*^2^, forces the predicted averages to be at the known distance *d*;
3. the regularization pushed the optimization to the minimal modification of the nominal model parameters **Θ**_n_ (Occam’s Razor). This ensures that the optimization converges to the most similar (yet calibrated) set of parameters to the nominal (ideal) model.

Since the optimization problem is nonlinear due to the forward kinematics model, it is common in the literature to initialize the model with the prior nominal model. However, if the nominal model is not provided by the manufacturer, the literature proposes methods to retrieve it, given the reading from the black-box controller^17^. On the other hand, for amateur projects, when the 3D CAD model is available, different tools can be used to generate the nominal geometric URDF model.

The proposed minimalist tool only has two sockets, different from other strategies that require three sockets^10^ or three spheres^7–9^. Two is the minimum number of sockets we can use. In fact, if only one socket is used, the optimization of the parameters may converge to any of the equivalent kinematic models that show consistent nullspace prediction but have proportionally bigger (or smaller) link dimensions. On the other hand, by having two sockets at a known distance, the optimization converges to the only set of link dimensions that ensures that our robot has high volumetric accuracy^18^ and high-nullspace consistency.

### Calibration assumptions

The uncertainty on the predicted Cartesian position is given by the uncertainty on the robot parameters Δ**Θ** (due to the manufacturing and assembly process) and on the joint angle Δ***q*** (due to noise in the encoder measurements or on the unobservable gear backlash and shaft flexibility). Nevertheless, we hypothesize the

#### Assumption 3

The uncertainty on the joint state ***q*** is negligible, i.e., Δ***q*** ≈ 0.

This implies that the observed Cartesian error, i.e., Δ***x***, is only due to the geometry uncertainty, i.e., Δ**Θ**. This is a fair assumption when using collaborative robots that use harmonic drive gears, which are known to have negligible backlash. However, this assumption is usually overlooked in other kinematic calibration methods, but it is a necessary condition to guarantee the convergence of the optimization.

To minimize the volumetric distortion of the method, the only ground truth measure that is exploited in this calibration routine is the distance between the two sockets and the minimal movement of the ball’s center point in a given socket. This brings us to

#### Assumption 4

The uncertainty on the socket distance d is negligible, i.e., Δ*d* ≈ 0. 

This assumption is valid if the manufacturing process has an uncertainty that matches the goal accuracy of the robot. Nevertheless, as depicted in Fig. 1, the tool is manufactured with two notches on the side of the sockets, designed to precisely place a caliper and measure the post-manufacturing distance with low uncertainty, thereby making Assumption 4 hold.

## Results

In order to validate the proposed tool and optimization methodology, we performed data recording and optimization for four different Franka Robots (three Panda and one FR3) with 7 DoF, one KUKA LBR iiwa 14 R820 with 7 DoF, and one Kinova Gen3Lite with 6 DoF.

The calibrated model was later used in a Cartesian impedance control^19^ for the Franka Robot to validate the calibrated model performance in high-precision insertion tasks or drawing retracing. The insertion and drawing were repeated from different joint configurations, demonstrating the calibrated model’s high consistency in insertion and retracing, in contrast to the original nominal model.

Moreover, it also performs the insertion on different known provided offsets on a calibrated table, showing that the distance tolerance matches the accuracy of 0.1 mm of the calibrated table. Volumetric calibration is particularly important when learned (or programmed) skills need to be generalized to different parts of the workspace. We 3D printed the two sockets at a distance of 50 mm and with a diameter of 20 mm, while the sphere was 19.9 mm. The employed 3-D printer is a commercial entry-level BambuLab A1 set to the extra fine accuracy, i.e., 0.08 mm. This is a very accurate, still affordable device.

### Calibrated model identification and validation

For any of the three families of robots to calibrate, we attached the spherical tool to the end-effector and positioned the two-socket tool in front of the robot. Setting the robots in a gravity-compensated and back-drivable modality allows for easy movement of the robot in different configurations with the spherical joint in the sockets. The users are asked to use the computer keyboard to add an observation or the buttons on the robots (available only on Franka) with a minimum of 30 samples per socket. One dataset, recorded on two sockets at a known distance, is sufficient for performing the kinematic calibration, which can be completed in a matter of minutes. However, in our study, we collected many datasets for each robot to test the consistency and distortion of the robot in different parts of the workspace. The unconstrained optimization problem (14) with *λ* = 10^−4^, is solved using an Interior Point Optimizer^20^, and if the relative change in the objective function is smaller than 10^−8^, the optimization is terminated. The exact first and second-order derivatives of the cost function with respect to the model parameters are obtained using the Automatic Differentiation engine of CasADi^21^. The cost function is the one reported in Eq. (14) that maximizes the consistency and minimizes the distortions while regulating the parameters to be as similar as possible to the nominal model. The displacement of the sphere relative to the last joint is initialized by performing the calibration while optimizing only this parameter. Moreover, the ball offset is excluded from regularization throughout any optimization since the initial guess may be significantly off.

**Fig. 3:**
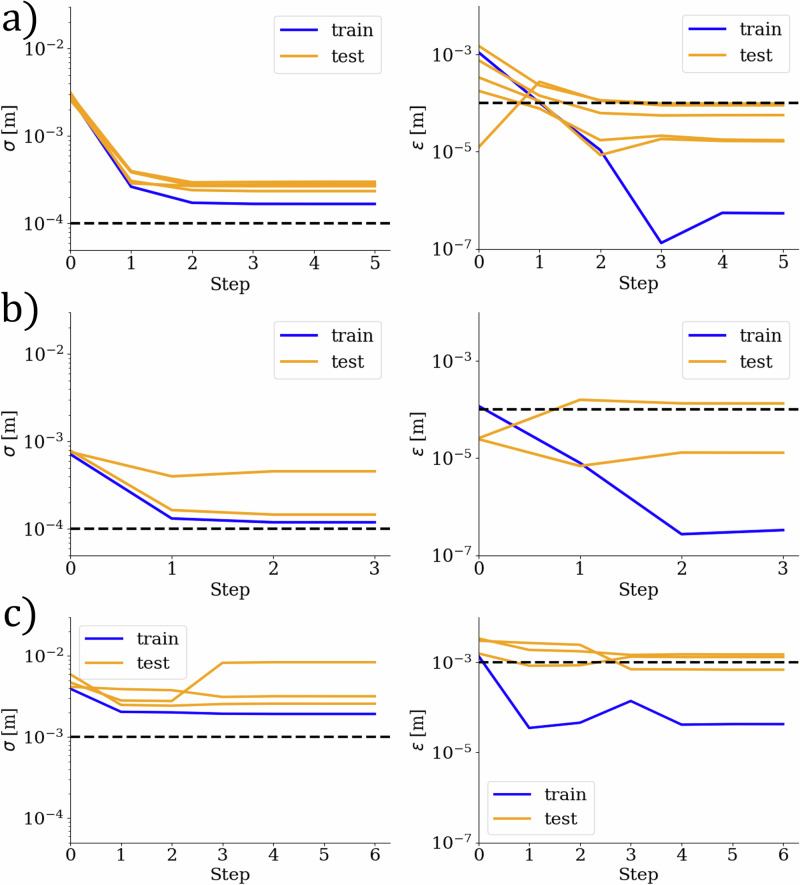
Optimization curves for different robotic platforms. **a** Franka Panda, **b** KUKA iiwa 14, and **c** Kinova Gen3 Lite. The plots show the convergence of the square root of the inconsistency (*σ*) and the distortion (*ϵ*) on training (blue) and test sets. See Eq. (14) for details. The dashed line depicts the robot repeatability declared by the manufacturer.

Figure 3a shows the optimization curve for a 7-DoF Franka robot. In a few iterations, the optimizer brings the error and the distortion from an order of magnitude of centimeters to submillimeters. This highlights that these robots are potentially very precise but the inaccuracy in their assembly generates a discrepancy between the nominal and the real model. The MUKCa tool closes the gap between the model and reality. Table 1 highlights the mean absolute error (MAE) with respect to the socket position estimated as the mean of the calibrated forward kinematics, i.e.

15 $$\frac{1}{({N}^{0}+{N}^{1})}\left(\mathop{\sum }\limits_{i=1}^{{N}^{0}}\parallel {\Phi }_{{{\boldsymbol{\Theta }}}}({{{\boldsymbol{q}}}}_{i}^{0})-{{{\boldsymbol{\mu }}}}^{0}\parallel +\mathop{\sum }\limits_{i=1}^{{N}^{1}}\parallel {\Phi }_{{{\boldsymbol{\Theta }}}}({{{\boldsymbol{q}}}}_{i}^{1})-{{{\boldsymbol{\mu }}}}^{1}\parallel \right)$$

where *Φ*_**Θ**_ is the forward kinematics parameterized with the calibrated parameters **Θ,** ${{{\boldsymbol{q}}}}_{i}^{0,1}$ are joints configurations that end up with the ball in socket 0 and 1 and ***μ***^0,1^ are the average prediction of the forward kineamtics in each socket.

**Table 1:** Mean Absolute Error (MSE) before and after the calibration on Franka, KUKA, and Kinova Robots

	Training Set		Test Set	
	Mean Absolute Error [m] (Orig. → Opt.)	Error reduction (%)	Mean Absolute Error [m] (Orig. → Opt.)	Error reduction (%)
Panda #1	8.28 × 10^−3^ → 1.68 × 10^−4^	97.97%	7.79 × 10^−3^ → 3.47 × 10^−4^	94.92%
Panda #2	3.32 × 10^−3^ → 1.51 × 10^−4^	95.43%	3.26 × 10^−3^ → 2.38 × 10^−4^	92.69%
Panda #3	8.79 × 10^−3^ → 1.84 × 10^−4^	97.91%	1.06 × 10^−2^ → 2.62 × 10^−4^	97.53%
FR3	8.51 × 10^−3^ → 1.61 × 10^−4^	98.11%	8.81 × 10^−3^ → 2.78 × 10^−4^	96.84%
KUKA iiwa 14	7.23 × 10^−4^ → 1.07 × 10^−4^	85.23%	8.06 × 10^−4^ → 2.68 × 10^−4^	66.76%
Kinova Gen3lite	4.72 × 10^−3^ → 1.52 × 10^−3^	67.77%	5.99 × 10^−3^ → 2.66 × 10^−3^	52.08%

**Orig**. refers to the original (or nominal) model, and **Opt**. refers to the optimized (or calibrated) model. The socket locations for the training and test set are defined in the Supplementary tables.

The MSE converges to values as little as 0.15 mm, deleting up to 98% of the original error, making the robot (almost) as accurate as repeatable. The dashed line depicts the repeatability of the robot reported by the manufacturer.

Similarly, Fig. 3b, shows the optimization curve for the KUKA iiwa 14. Given a more accurate assembly process, the original nominal model already exhibits a submillimetric precision ( ≈ 0.7 mm). Nevertheless, by using the calibration tool, the error reduces to 0.107 mm, matching the robot’s repeatability.

However, approaching submillimetric accuracy is not possible for every robot, in particular, if the assumption of negligible joint uncertainty is not valid due to backlash. This is the case of Fig. 3c for the Kinova Gen3lite: the calibration converges to an average of 2 mm starting from an accuracy of 5 mm. However, since the estimated repeatability of the robot is around 1 mm, the calibration routine still managed to correct all the geometric inaccuracies.

From the experiment, we conclude that robots that have harmonic drives, like the Franka and KUKA, result in very precise calibration. On the other hand, when calibrating robots that use planetary gearboxes, like the Kinova, the calibration did not have the same miraculous improvements. We identify the problem to be in the backlash in the joints, which is not observable in the encoder measurements. There is an active effort in the robotics community for the parameterization^18^ and the compensation of the backlash^22^ that can complement the research of this paper in decreasing the inaccuracy of cheaper robots.

**Fig. 4:**
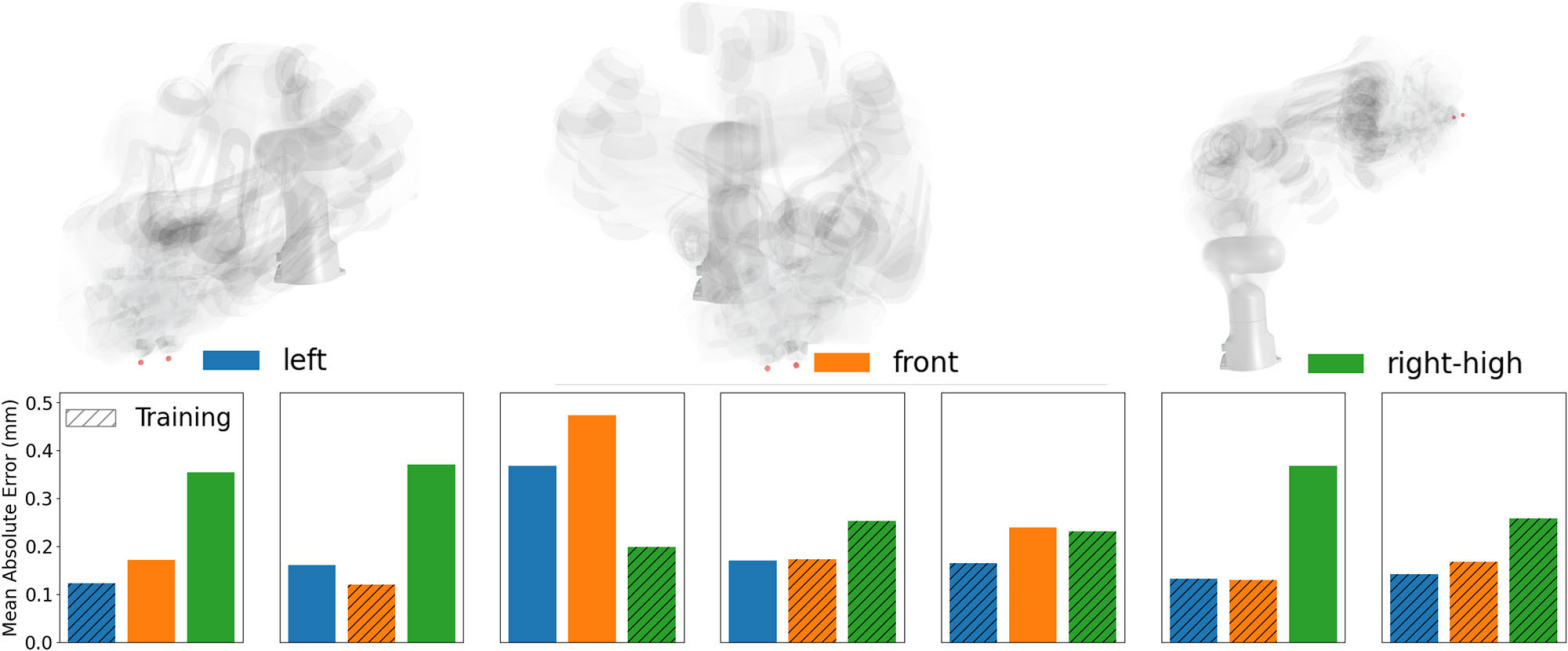
Converging mean squared error when training on multiple tool positions. The overlay shows the prediction after training for the tool. The hashed bar implies that the data for that tool was observed during training. The red dots are the overlapping prediction of the ball center point in different configurations.

Although training the robot in a single tool position is shown to be sufficient to reduce error and improve consistency globally, we observe that accuracy slightly degrades for tool positions farther from the training position. This phenomenon is analyzed and quantified in Fig. 4. We recorded three widely separated datasets for a Franka robot: one in front of the table, one to the extreme left at table height, and one on the right at a higher position attached to the wall. We trained on each position individually and tested on the others, trained on two positions and tested on the remaining one, and finally trained on all positions simultaneously. The results show that training on the front position yields similar accuracy for the left position but worse accuracy for the higher position. It is worth mentioning that reaching an accuracy error of 0.3 mm for a faraway position is still an impressive result considering the original 15 mm error. However, even when training exclusively in the high position, the error does not converge to the same small quantity as when training in the frontal position. The comparisons suggest that the best overall accuracy is obtained by training on the two most distant tool positions in the dataset, or, if only one position is available, prioritizing the frontal positions.

### Ablation study and comparisons

**Fig. 5:**
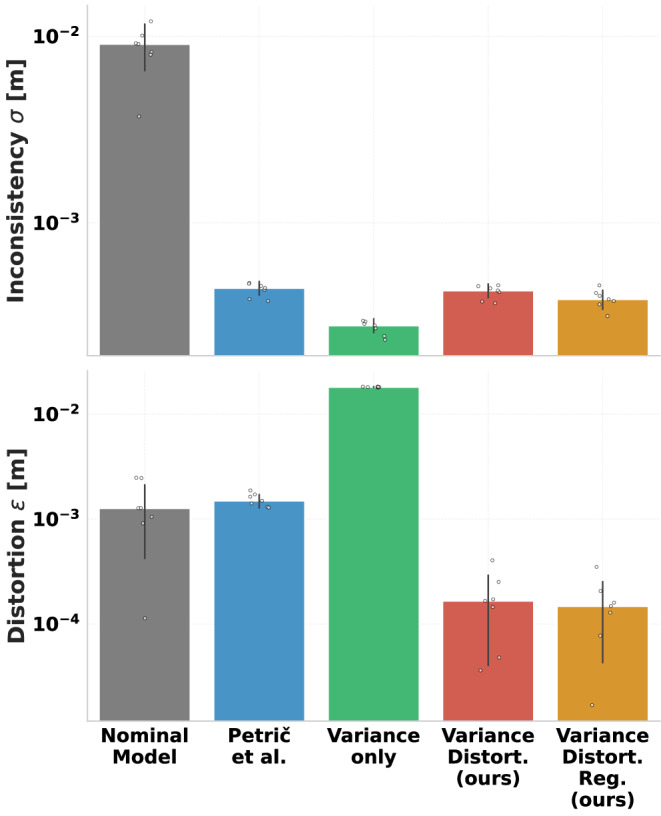
Ablation study and comparison with Petric et al.^10^ on test data of Panda #1. Our approach achieves lower inconsistency and distortion, regardless of whether model regularization is used or not. The bars are the average value for distortion and inconsistency defined in Eq. (14). Lower values are better.

To highlight the effect of different terms of the cost function, Fig. 5 depicts the convergence on the test set for both consistency and distortion when using, in Eq. (14), only the variance, variance and distortion and finally adding the regularization term. It also benchmarks an alternative state-of-the-art method^10^ that minimizes MSE with respect to the predicted average of the nominal model. The bar plot highlights how Petrič et. al.^10^ increases the nullspace consistency but retains distortion in the model. Similarly, by only minimizing variance, we obtained a consistent model but with an increased volumetric distortion. The only way of obtaining a consistent and undistorted method is to minimize the distortion (or bias).

Moreover, we notice that the regularization acts as an extra safeguard layer that does not have a clear impact on the optimization in this case. To tune it, a good rule of thumb is to start from a very low regularization and only increase it if converging to a dysmorphic URDF.

### Robot validation: insertion tasks and motion retracing

To validate the improved performance of the calibrated model obtained using the proposed MUKCa procedure, we perform vertical insertion tasks with a peg of 10 mm diameter attached to the end-effector and holes with different dimensions, from 16 mm down to 10.4 mm. The goal is to validate the *consistency* of the model when performing the insertion task by starting from different nullspace configurations and the *distortion* of the model when offsetting the hole by a known distance on a calibrated table. The experiment was performed on the Franka robot controlled with a Cartesian impedance controller.

**Fig. 6:**
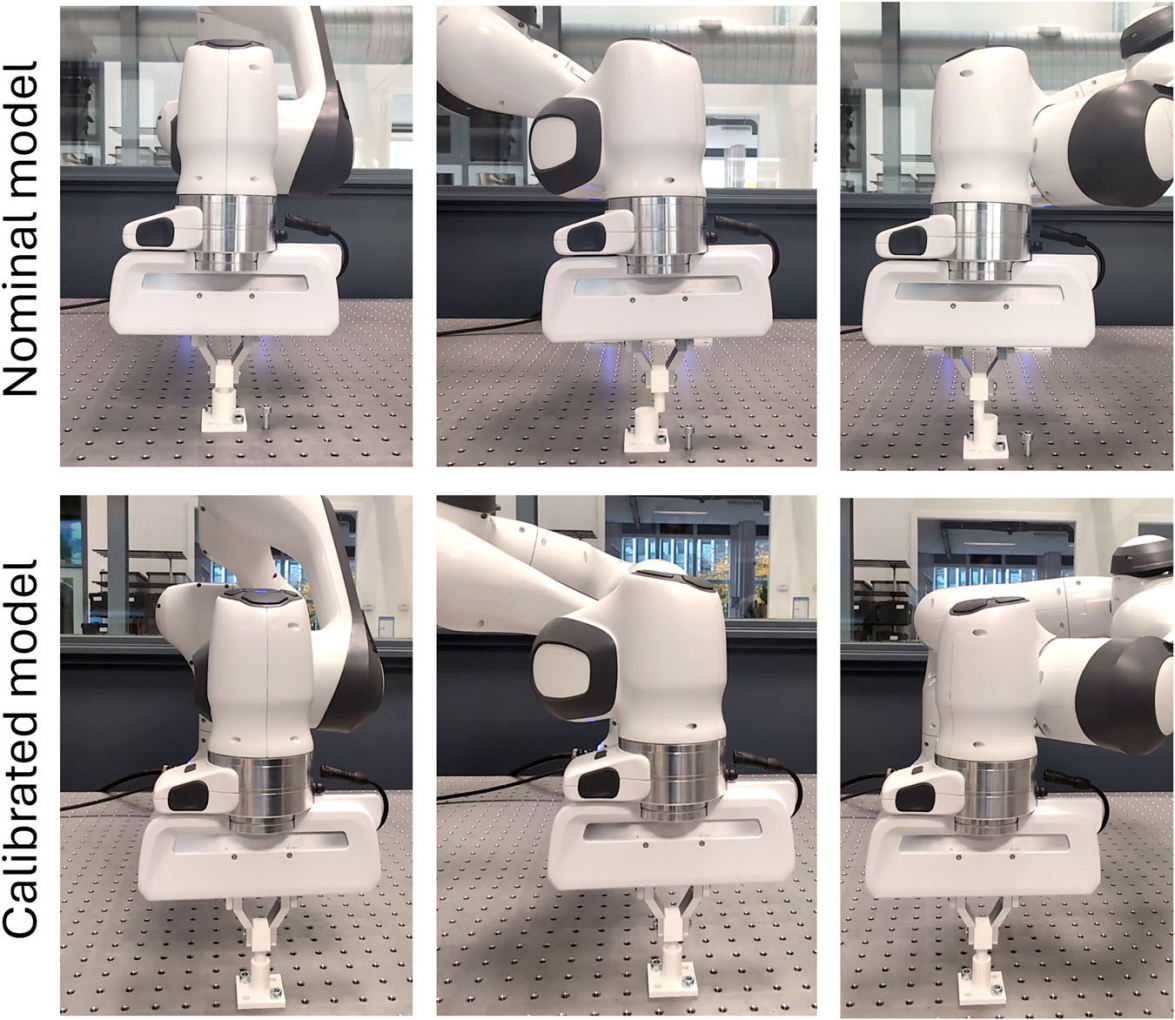
Insertion task with the nominal model or calibrated model using Panda #1. After calibration, the robot successfully inserts the peg (10 mm) in the hole (10.4 mm) despite the changes in the initial starting joint configuration. The nominal model even fails with a hole of 16 mm.

Performing consistent insertion tasks with different joint configurations is a necessary feature of redundant manipulators. Figure 6 illustrates the test bench of the performed *consistency* test with a hole of 16 mm of diameter using the nominal model provided by the manufacturer. The goal pose is computed by manually positioning the robot, with the elbow up, in a tight hole and recording the desired Cartesian pose from the forward kinematics. However, when executing the insertion task again with a different starting joint configuration, the resulting insertion fails despite the large tolerance between the peg and the hole. It is worth explaining that the reason for the failure is not due to controller compliance or manipulability singularities. In fact, we employ high stiffness and compensate for static friction before the insertion, see the supplementary notes 1. Before the insertion, the robot erroneously *believes* that the Cartesian error is very small. This underlines that the issue is the *miscalibration* of the forward kinematics model.

To prove this, we repeated the experiment using the calibrated forward kinematics and Jacobian model computed using Pinocchio^23^ and the calibrated URDF model. Figure 6 shows the insertion performance of a hole of 10.4 mm. The insertion was performed 20 times by randomizing the starting joint configuration, and it always resulted in a successful insertion.

Moreover, to validate the volumetric calibration of the new model and that the robot is not only locally accurate^24^, the hole was moved to 24 other positions on an “X” shape (6 on each branch) on a calibrated table. The screws are at a distance of 25 mm in the x and y axes with an accuracy of 0.1 mm. The x-y desired insertion coordinate is displaced according to the movement of the peg on the table. The experiment resulted in all successful 24 insertions, however, with a lower or higher interaction force due to the inaccuracy of the peg/hole manufacturing, kinematic model, and calibrated table, with a mean force of 6 N and max force of 30 N and min of 0.7 N.

**Fig. 7:**
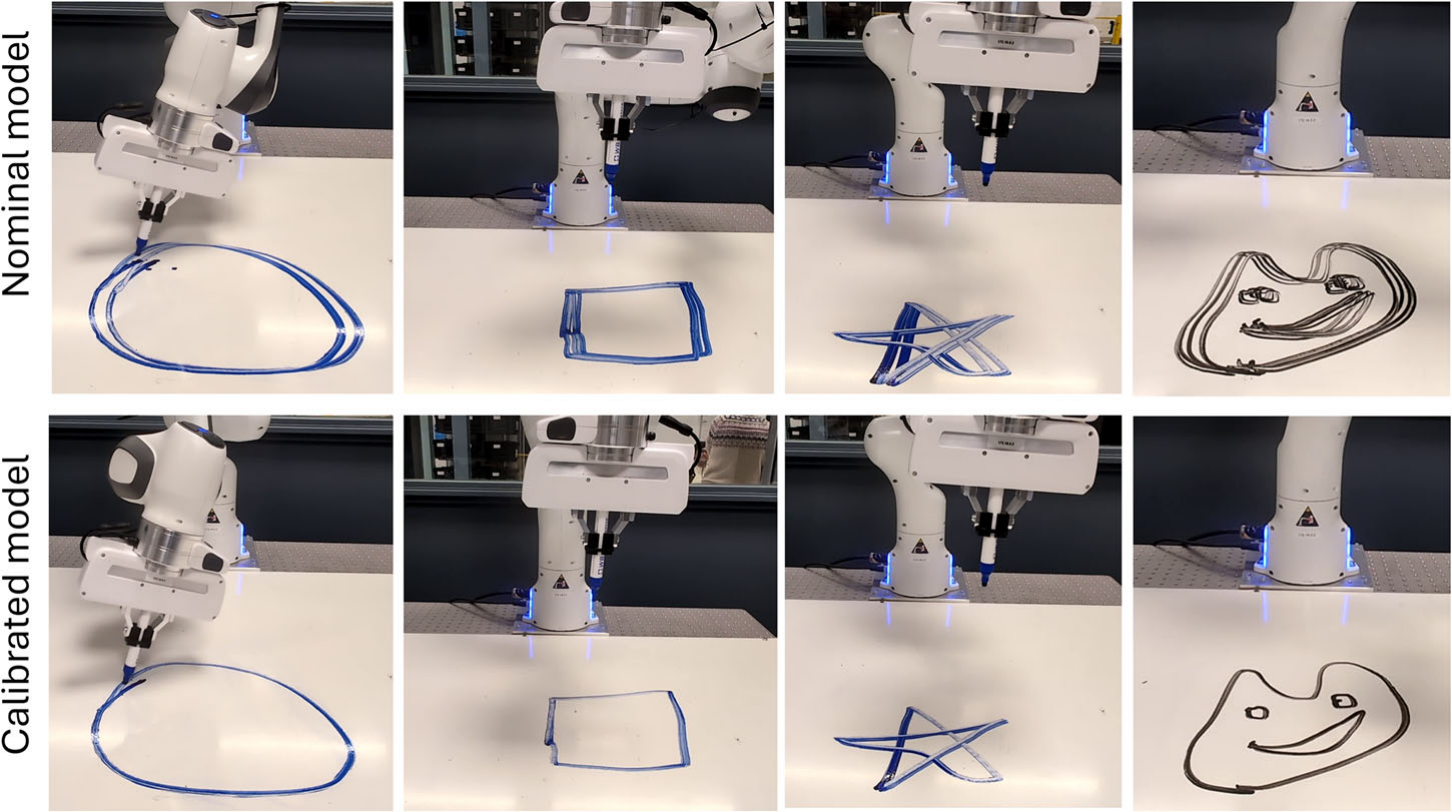
Accuracy of learned drawing motion using Panda #1. By executing the Cartesian motion with different joint evolutions, the calibrated models perfectly trace the previous iterations. The nominal model clearly fails on the task.

Finally, we also recorded a drawing motion using kinesthetic teaching and executed it using the calibrated or the nominal model. Figure 7, depicts the attempt of the retracing of the same Cartesian motion starting from a different joint configuration. The figure highlights how the nominal model terribly fails at the task while the calibrated model consistently retraces the same original drawing.

## Conclusion

This paper introduces and formalizes a novel calibration tool and optimization routine that only relies on the minimization of the nullspace inconsistency for each of the two sockets and the bias or distortion of the predicted distance between the averages. We only require three Cartesian quantities to ensure the convergence of the calibration. The only advantage of using three spheres^9^ (or sockets^10^) is three additional independent quantities (two distances and one consistency), improving numerical stability, reducing sensitivity to measurement noise, and ensuring more robust and accurate parameter estimation in the optimization process. However, in this article, we experimentally validated that the minimal hardware with two sockets is enough for obtaining accurate calibration.

Despite the simplicity and the affordability of the device that is fully 3D printable, the mean absolute error was reduced from the order of 1 cm to the order of 0.1 mm for a Franka robot. This was also validated by performing high-precision insertion tasks before and after the calibration, showing the improvement from failing insertion tasks with 6 mm tolerance to repeatedly inserting the peg in the hole with only 0.4 mm clearance. However, we identify the limitation in calibrating robots that do not guarantee a minimal joint measurement error, like the Kinova Gen3lite, due to backlash and/or sensor noise. We even observed that performing the calibration routine on these robots can result in overfitting, i.e., an increasing error for positions further away from the tool. Future work should focus on applying the methodology to cheaper robots that are not equipped with harmonic drivers or double encoders^25^, or are not utilizing the torque sensor to estimate and compensate for backlash^26,27^ or joint deformation^28^. We foresee exciting challenges and opportunities in the simultaneous calibration of robot geometry, backlash, and joint deformation by only using a simple 3D-printable artifact, as in this paper.

## Supplementary information

Transparent Peer Review file

Supplementary Material

## Data Availability

The data recorded for the experiments are different sets of joint configurations for each socket and are publicly available at https://github.com/platonics-delft/kinematics_calibration/tree/main/data.

## Code availability

The code is publicly available at https://github.com/platonics-delft/kinematics_calibration.
