# Supplementary material for: Accurate and affordable cobot calibration without external measurement devices
Source: Commun Eng. 2026 Apr 6;5:138. doi: 10.1038/s44172-026-00633-4 (PMC13421692; doi:10.1038/s44172-026-00633-4)
Supplement: Supplementary file 2 — Supplementary Material [file 44172_2026_633_MOESM2_ESM.pdf]

Supplementary Notes 1

Impedance control is crucial in robot manipulation because it enables robots to interact with their environments in a flexible, adaptive, and safe manner. Traditional position or force control methods can be too rigid, resulting in failures or damage when dealing with unpredictable or dynamic environments. This control allows robots to modulate the relationship between applied force and the resulting motion, mimicking human-like adaptability in tasks requiring both precision and delicacy, such as assembly, object handling, or interaction with humans. By dynamically adjusting its stiffness, the robot can control the accuracy in the performing task. This adaptability makes impedance control particularly valuable for tasks that involve physical contact, ensuring safer human-robot collaboration and more effective execution of insertion and fine manipulation tasks.

The dynamic equation of a robot manipulator is defined according to

$$\boldsymbol{\mathcal{M}}(\boldsymbol{q})\ddot{\boldsymbol{q}} + \boldsymbol{\mathcal{C}}(\boldsymbol{q}, \dot{\boldsymbol{q}}) + \boldsymbol{\mathcal{G}}(\boldsymbol{q}) = \boldsymbol{\tau}_{\mathrm{c}} + \boldsymbol{\tau}_{\mathrm{comp}} + \boldsymbol{\tau}_{\mathrm{ext}} \tag{16}$$

where, in order, from left to right, there are the mass, the Coriolis, and the gravitational term that depend on the joint configuration $\boldsymbol{q}$ and, on the right, the torque for the Cartesian (or task) control, the torque that compensates for frictions and other unmodelled dynamics and the externally applied torques. The task space torque is computed as

$$\boldsymbol{\tau}_{\mathrm{c}} = \boldsymbol{J}_{\boldsymbol{\Theta}}^{\top}(\boldsymbol{\mathcal{K}}\left(\boldsymbol{x}_{\mathrm{goal}} - \Phi_{\boldsymbol{\Theta}}(\boldsymbol{q})\right) - \boldsymbol{\mathcal{D}}(\boldsymbol{J}_{\boldsymbol{\Theta}}\dot{\boldsymbol{q}})) + \boldsymbol{\mathcal{C}}(\boldsymbol{q}, \dot{\boldsymbol{q}}) + \boldsymbol{\mathcal{G}}(\boldsymbol{q}) \tag{17}$$

where the stiffness $\boldsymbol{\mathcal{K}}$ and the damping $\boldsymbol{\mathcal{D}}$ give the compliant behavior with a critically damped response.[18] The error from the desired pose $\boldsymbol{x}_{\mathrm{goal}}$ is computed using the forward kinematics $\Phi_{\boldsymbol{\Theta}}(\boldsymbol{q})$. The projection of the Cartesian forces in joint torques is obtained using the transpose of the geometric Jacobian $\boldsymbol{J}_{\boldsymbol{\Theta}}$. Both the Jacobian and the forward kinematics depend on the model parameters $\boldsymbol{\Theta}$. From Eq. (17), it is evident that relying on a miscalibrated model for control is a recipe for disaster, accumulating errors in the position tracking, the Cartesian velocity estimation, and the force-torque projection.

Moreover, since we are testing high-precision tasks, no residual error due to the unmodelled static friction in the joints is acceptable. Without compensating the static friction, not even a perfectly calibrated robot can perform a sub-millimetric insertion task because the tracking error would be larger than the insertion tolerance, i.e., $\epsilon_{\mathrm{trac}} = |(\boldsymbol{x}_{\mathrm{goal}} - \Phi_{\boldsymbol{\Theta}}(\boldsymbol{q})| > \epsilon_{\mathrm{tol}}$, making a perfectly calibrated model incapable of performing tight insertion tasks.

To address this problem, just before the insertion task, an integral compensator is activated for a time interval Δt, i.e.,

$$\tau_{\mathrm{comp}} = \mathcal{K} \int_{0}^{\Delta t} \left(\boldsymbol{x}_{\mathrm{goal}} - \Phi_{\boldsymbol{\Theta}}(\boldsymbol{q})\right) dt. \tag{18}$$

and set to zero otherwise. The compensator is not activated during the insertion task itself, where there are external forces due to the contact between the peg and the hole. Having the compensator may result in winding up the force that can generate instabilities and/or damage the tool settings.

For a perfectly compensated controller,

$$\boldsymbol{x}_{\mathrm{goal}} - \Phi_{\boldsymbol{\Theta}}(\boldsymbol{q}) \approx 0; \tag{19}$$

however, the actual robot position $\boldsymbol{x}$ is

$$\boldsymbol{x} = \Phi_{\boldsymbol{\Theta}}(\boldsymbol{q}) + \epsilon_{\mathrm{cal}}(\boldsymbol{q}) \tag{20}$$

where the $\epsilon_{\mathrm{cal}}(\boldsymbol{q})$ is the residual error that we aim to reduce with the calibration procedure. By combining the two equations,

$$\boldsymbol{x}_{\mathrm{goal}} - \boldsymbol{x} + \epsilon_{\mathrm{cal}}(\boldsymbol{q}) \approx 0 \tag{21}$$

showing that the observed tracking error is not due to the control or the unmodelled frictions but to the kinematics miscalibration, i.e.,

$$\underbrace{|\boldsymbol{x}_{\mathrm{goal}} - \boldsymbol{x}|}_{\epsilon_{\mathrm{tracking}}} \approx |\epsilon_{\mathrm{cal}}(\boldsymbol{q})|. \tag{22}$$

Supplementary Tables

Table II reports the used parameters in the experiments on Robot Validation. Table III summarizes the tool locations for the training and the testing relative to the performance evaluation for Table I in the main text.

$\mathcal{K}_x$	$\mathcal{K}_y$	$\mathcal{K}_z$	$\mathcal{K}_\alpha$	$\mathcal{K}_\beta$	$\mathcal{K}_\gamma$	$\mathcal{D}$	Δt[s]
15000	15000	2000	40	40	40	$2\sqrt{\mathcal{K}}$	5

TABLE II: Experimental parameters used in the robot validation. The linear stiffness is measured in $\mathrm{N/m}$ and the rotational one in $[\mathrm{Nm/rad}]$

	**Training Sockets**	**Test Sockets**	
**Robot**	**Socket 0 and 1**	**Socket 0**	**Socket 1**
	0.477 -0.022 0.016	0.503 -0.273 0.016	0.503 -0.223 0.016
	0.476 0.028 0.016	0.252 -0.249 0.016	0.251 -0.199 0.016
		-0.002 0.277 0.016	-0.002 0.327 0.016
		0.343 0.404 0.100	0.379 0.403 0.136
Panda #1		0.475 0.229 0.016	0.475 0.278 0.017
		0.001 -0.326 0.016	0.001 -0.276 0.016
		0.250 0.228 0.016	0.249 0.278 0.016
	0.625 -0.025 0.016	0.476 -0.025 0.016	0.476 0.025 0.015
Panda #2	0.626 0.025 0.016	0.476 0.150 0.015	0.476 0.200 0.016
		0.476 -0.200 0.016	0.476 -0.150 0.016
		0.626 0.150 0.016	0.626 0.200 0.016
		0.626 -0.225 0.016	0.626 -0.175 0.016
	0.419 -0.027 0.020	0.522 -0.226 0.020	0.521 -0.176 0.020
Panda #3	0.418 0.023 0.020	0.465 0.225 0.021	0.464 0.275 0.021
	0.401 -0.029 0.017	0.399 -0.230 0.017	0.400 -0.180 0.017
FR3	0.401 0.021 0.017	0.526 -0.030 0.018	0.527 0.020 0.018
	0.551 -0.026 0.009	0.437 -0.232 0.506	0.437 -0.232 0.556
Kuka	0.551 0.024 0.009	0.300 -0.452 0.009	0.300 -0.402 0.009
	0.030 -0.230 0.081	0.243 -0.071 0.206	0.239 -0.069 0.155
Kinova	-0.020 -0.228 0.082	0.254 0.084 -0.025	0.252 0.135 -0.022
		0.091 0.135 0.083	0.091 0.186 0.084

TABLE III: Training and test socket positions for each of the calibrated robots in this study.
