# Supplementary material for: Accurate and affordable cobot calibration without external measurement devices
Source: Commun Eng. 2026 Apr 6;5:138. doi: 10.1038/s44172-026-00633-4 (PMC13421692; doi:10.1038/s44172-026-00633-4)
Supplement: Supplementary file 1 — Transparent Peer Review file [file 44172_2026_633_MOESM1_ESM.pdf]

nature portfolio

Peer Review File

MUKCa: Accurate and Affordable Cobot Calibration Without External Measurement Devices

Corresponding Author: Dr Giovanni Franzese

This file contains all reviewer reports in order by version, followed by all author rebuttals in order by version.

Version 0:

Reviewer comments:

Reviewer #1

(Remarks to the Author)
See the attached file.

Reviewer #2

(Remarks to the Author)
This paper presents a cost-effective yet efficient alternative to existing calibration schemes, utilizing a 3D-printed tool as the sole physical aid in the calibration process. This ball-joint tool, constrained by dual sockets, applies kinematic constraints to the robot's end-effector for training data collection. Model parameter optimization is achieved by reducing the standard deviation of the data within each socket and minimizing the deviation between the measured distance of the two sockets and the known distance. The method was validated on Franka, Kuka, and Kinova Cobots: calibration reduced the mean absolute error from 10 mm to 0.2 mm for Franka and Kuka robots. Additional experiments on the Franka robot verified the method's feasibility: 1) Performing a peg-in-hole task (allowable peg-hole clearance: 0.4 mm) using Cartesian control before and after calibration. 2) Executing a repetitive drawing task by combining Cartesian control with teaching. Results showed that both tasks consistently failed without calibration but consistently succeeded after calibration.
The reviewer believes the following aspects require supplementation before further decision:
1. In Section I, the authors emphasize that the proposed method " eliminating the limitation of relying on the average nominal model " but the specific limitations are not clearly defined. The experimental section should also include comparative experiments with this method to demonstrate the superiority of the proposed approach.
2. Equation (5) in Section III.C is the proposed cost function, consisting of inconsistency, distortion, and regularization terms. The first and second terms include the mean value (μ). This appears to contradict the emphasis in Section I regarding eliminating reliance on the average. The authors need to further clarify how this effectively removes the limitation of dependence on the average.
3. In Section III.C, the authors state: " To minimize the volumetric distortion of the method, the only ground truth measure that is exploited in this calibration routine is the distance between the two sockets and the minimal movement of the ball’s center point in a given socket." This highlights the critical importance of the MUKCa calibration tool's manufacturing precision and reliability. However, the device is solely 3D-printed. Concerns are raised regarding factors such as printing material, manufacturing accuracy, potential shifts in the ball center point during human operation, and tool deformation under force. The authors should supplement relevant content to demonstrate the reliability of the MUKCa calibration tool.
4. The parameter description and conclusion analysis in Section IV.A need further enrichment and strengthening. It is suggested to supplement information such as the number of data points collected per socket and the distance between the sockets. For Figure 6, it is recommended to clearly specify the number of data points used for training or prediction at different workpiece positions and supplement relevant theoretical analysis.
5. In Section IV.B, the authors use a 10 mm diameter peg to perform peg-in-hole operations on holes ranging from 16 mm to 10.4 mm diameter to verify model consistency. The allowable clearance varied from 6 mm to 0.4 mm. While the experimental results effectively demonstrate the feasibility of the proposed method, its superiority requires comparative validation against state-of-the-art alternative methods.
6. Section IV.B mentions: "The goal pose is computed by manually positioning the robot, with the elbow up, in a tight hole and recording the desired Cartesian pose from the forward kinematics." The authors first teach a robot configuration for peg insertion (where insertion error is approximately zero) and then execute the peg-in-hole task 20 times at the same location with random configurations. Due to positional error similarity [1], it is suggested to supplement the distribution of robot

configurations during the peg-in-hole task execution and use quantitative peg insertion error instead of the qualitative description "peg-in-hole success or failure." The drawing task description should undergo the same modification. This will enhance the persuasiveness and rigor of the paper.
[1] Zeng Y, Tian W, Liao W. Positional error similarity analysis for error compensation of industrial robots[J]. Robotics and Computer-Integrated Manufacturing, 2016, 42: 113-120.
7. The method used in Section IV for robot accuracy verification differs from conventional large-workspace robot accuracy validation based on laser trackers. Please justify the rationality of the experimental verification approach.

Reviewer #3

(Remarks to the Author)
This paper introduces MUKCa, which uses only a 3D-printed two-socket spherical-joint tool to constrain the end-effector during data collection and an optimization routine that minimizes socket-position inconsistency and model distortion. The method is experimentally validated on Franka, KUKA, and Kinova robots, achieving significant accuracy improvements . The authors further demonstrate the benefits in high-precision tasks, including peg-in-hole insertion and drawing via kinesthetic teaching, showing consistent success after calibration. However, the following comments shall be considered:

(1)While the method works very well for robots with Franka, KUKA, the performance degrades significantly on the Kinova robot. This limitation is acknowledged but could be better quantified and discussed in terms of applicability boundaries.

(2)The paper notes that calibration accuracy degrades at positions far from the training location, especially with low-cost robots. While additional datasets improve performance, a more systematic analysis of workspace coverage requirements would be valuable.

(3)The approach depends on several assumptions (back-drivable motors, negligible encoder noise, negligible socket distance uncertainty). These are reasonable for many cobots but may not hold universally. The sensitivity to violations of these assumptions should be more explicitly evaluated.

(4)Please provide one figure in Section III for better understanding, as in the literature: Z. Fu, E. Spyrakos-Papastavridis, Y. -h. Lin and J. S. Dai, "Analytical Expressions of Serial Manipulator Jacobians and their High-Order Derivatives based on Lie Theory," 2020 IEEE International Conference on Robotics and Automation (ICRA), Paris, France, 2020, pp. 7095-7100, doi: 10.1109/ICRA40945.2020.9197131

Version 1:

Reviewer comments:

Reviewer #1

(Remarks to the Author)
The authors have addressed all of my concerns and provided appropriate clarifications and revisions where necessary. The revisions have improved the quality and clarity of the manuscript. In light of these improvements, I am pleased to recommend that the manuscript be accepted for publication in its current form.

Reviewer #2

(Remarks to the Author)
The authors have addressed all my comments.

Reviewer #3

(Remarks to the Author)
All my concerns have been addressed.

Open Access This Peer Review File is licensed under a Creative Commons Attribution 4.0 International License, which permits use, sharing, adaptation, distribution and reproduction in any medium or format, as long as you give appropriate credit to the original author(s) and the source, provide a link to the Creative Commons license, and indicate if changes were made.
In cases where reviewers are anonymous, credit should be given to 'Anonymous Referee' and the source.
The images or other third party material in this Peer Review File are included in the article's Creative Commons license, unless indicated otherwise in a credit line to the material. If material is not included in the article's Creative Commons license and your intended use is not permitted by statutory regulation or exceeds the permitted use, you will need to obtain permission directly from the copyright holder.
To view a copy of this license, visit https://creativecommons.org/licenses/by/4.0/

MUKCa: Accurate and Affordable Cobot Calibration Without External Measurement Devices

Giovanni Franzese, Max Spahn, Jens Kober, Javier Alonso-Mora, and Cosimo Della Santina

RC: Reviewer Comment, AR: Author Response, ☐ *Manuscript text*

Reviewer 1

RC: **However, while the concept of low-cost calibration is commendable, the work fails to demonstrate significant advantages over existing approaches, particularly the closely related reference [1] (Petričet al.). The claimed contributions—eliminating the need for a third socket and avoiding reliance on nominal model to obtain the average positions of sockets — do not translate to measurable performance improvements. Inadequate presentation issues further undermine the paper's validity. The manuscript reports that MUKCa reduces the mean absolute error (MAE) for Franka robots to 0.15–0.3 mm (Table I and Section IV.A). However, as shown in the last subfigure of Fig. 6, Petri's three-socket method [1] achieves comparable accuracy (0.2–0.3 mm) on similar Franka robots when trained simultaneously across all positions. This marginal difference ($< 0.05mm$) is within typical experimental variability, offering no meaningful improvement in precision. The overlap in performance undermines the claim that MUKCa represents a significant advance. The authors emphasize the affordability and simplicity of a two-socket tool, but this advantage is negligible in the context of offline calibration. Both MUKCa and [1] require offline data collection (30+ samples per socket), and the incremental cost or complexity of adding a third socket (especially for 3D-printed tools) is minimal.**

AR: We thank the reviewer for highlighting the weak point. With MUKCa, our goal was to find the minimal tool and optimization routine that a final user would need to calibrate their robot. In the text, we highlight that our approach is closely related to [1], still a completely new definition of the optimization paradigm. We are not optimizing the mean square error with respect to the average value of the original model for each socket. We highlight that the approach of [1] cannot fix the volumetric distortion of the original model. To show this, we compare the optimization when using the mean square error vs the variance plus bias approach proposed in this article. We added a new box plot in the text, also reported here for simplicity in Figure 1. The reviewer would notice that the optimization in Petric et al. converges to a very low variance in the prediction but retains distortions in the model since it computes the target for the MSE using the (distorted) nominal model. On the other hand, the variance plus bias paradigm that we introduced fixes the issue. The paper does not only contributes with a simpler device that can be easily 3D printed. But also highlights how, when dealing with a non-external measurement device, the only ground truth is the socket consistency and the known distance, without relying on the nominal model to generate the average ground truth. We only rely on the nominal model to regularize the optimization and safeguard it from converging to dimorphic URDFs.

To highlight the effect of different terms of the cost function, Figure 1 depicts the convergence on the test set for both consistency and distortion when using only the variance, variance and distortion and finally adding the regularization term and compares with an alternative state of the art method [1] that minimizes MSE with respect to the predicted average of the nominal model. The box plot highlights how this method increases the nullspace consistency but retains distortion in the model. Similarly, by only minimizing variance, we obtained a very consistent model but with an increasing distortion. The only way of obtaining a consistent and undistorted method is to minimize the distortion (or bias).

Moreover, we notice that the regularization acts as an extra safeguard layer that does not have a clear impact on the optimization in this case. To tune it, a good rule of thumb is to start from a very low value and only increase it if converging to a dimorphic URDF.

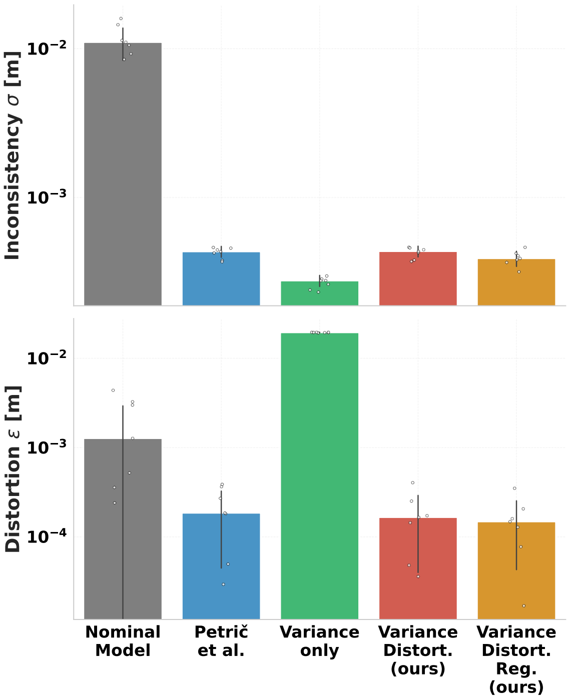

Figure 1: Comparison with Petric and ablation study

A careful reader or reviewer might wonder whether the observed issue stems from an improper initialization of the sphere (or ball) offset in the model. To prevent this, we initialize the ball parameters by solving an optimization problem that adjusts only this specific set of parameters. Consequently, when the full optimization begins, any residual error can be attributed solely to the miscalibration of the kinematic chain. We also added this in the text:

The displacement of the sphere relative to the last joint is initialized by performing the calibration while optimizing only this parameter. Moreover, the ball offset is excluded from regularization throughout any optimization.

RC: **In Section IV.A, the authors describe MUKCa uses random test positions "many datasets... in different parts of the workspace" without clear partitioning. As is well-established in robot calibration, test data results will approximate training data performance when the test set closely resembles the training distribution. This principle necessitates spanning the entire workspace with training data during calibration. When comparing methods in Petrič [1], the authors explicitly evaluate calibration generalization by structuring training and testing datasets across defined workspace regions: "one in front of the table, one to the extreme left at table height, and one on the right." MUKCa does not adopt this protocol, instead using random cross-validation. This comparison is neither fair nor rigorous.**

AR: We thank the reviewer for highlighting this. We agree that omitting the position of the training socket and the evaluation socket made the first version of the manuscript weaker. In supplementary materials, we added a table where all the points for the training and the evaluation of Table 1 of the main text are specified.

Table 1 summarizes the tool locations for the training and the testing relative to the performance evaluation of Sec. II A and Table I.

Moreover, we believe that showing the use of the calibrated model in the Cartesian control of the robot, showing accurate insertions and repeatable drawing, is an impactful way of model validation, beyond the simple numerical investigation. For example, we carefully evaluated the nullspace consistency in the insertion by randomizing the initial joint configuration. Moreover, we also moved the hole in the insertion task to another 24 positions on a table at a known distance (by using a calibrated table). We showed that the model was correctly calibrated since the requested movement in the model, resulted in the precise movement on the robot and successful insertion. To quote the text:

Moreover, to validate the volumetric calibration of the new model, the hole was moved on 24 other positions positioned on an "X" shape (6 on each branch) on a calibrated table. The screws are at a distance of $25\,\mathrm{mm}$ *in the x and y axis with an accuracy of* $0.1\,\mathrm{mm}$. *The x-y desired insertion coordinate is displaced according to the movement of the peg on the table. The experiment resulted in all successful 24 insertions, however, with a lower or higher interaction force due to the inaccuracy of the peg/hole devices, kinematic model, and calibrated table, with a mean force of* $6\,\mathrm{N}$ *and max force of* $30\,\mathrm{N}$ *and min of* $0.7\,\mathrm{N}$.

RC: In the abstract, the authors claim "... reduce the mean absolute error from the order of 10mm to 0.2mm for both Franka and Kuka robots." However, TABLE I shows that the result of mean absolute error for Kuka robot before calibration is 7.23 × 10-4 (training set) or 8.06 × 10-4 (test set). Although the authors do not specify the unit in TABLE I, based on the context, the unit in this table should be meters. The mean absolute error for Kuka robot, either Abstract or TABLE I, one of them must be incorrect.

AR: We thank the reviewer for highlighting the problem. We agree that the abstract was not formulated correctly. We now mainly highlight the remarkable reduction of error of the Franka in the abstract, removing any kind of ambiguity. The Kuka robot is by default more accurate; however, after calibration, the robot's precision matches the robot's repeatability.

We added the missing unit of measure in the table.

RC: The variance-bias decomposition of mean squared error (MSE) in Section III.C is central to MUKCa's cost function but lacks supporting references. This decomposition is not usual to kinematic calibration, and readers require citations to foundational work (e.g., statistical learning literature on bias-variance tradeoffs) or an appendix with derivations to validate Its application here.

AR: We thank the reviewer for pointing out the missing reference and for highlighting the novelty of our approach in the field of kinematic calibration. In the text now we add:

By using the variance-bias decomposition [2] of each of the two mean squared error costs, [...]

The reference specifies the pages where the variance-bias decomposition of error is formalized. We agree that this may help future readers to better grasp the proposed decomposition of this article.

	Training Sockets	Test Sockets	
**Robot**	**Socket 0 and 1**	**Socket 0**	**Socket 1**
Panda #1	0.477 -0.022 0.016	0.503 -0.273 0.016	0.503 -0.223 0.016
	0.476 0.028 0.016	0.252 -0.249 0.016	0.251 -0.199 0.016
		-0.002 0.277 0.016	-0.002 0.327 0.016
		0.343 0.404 0.100	0.379 0.403 0.136
		0.475 0.229 0.016	0.475 0.278 0.017
		0.001 -0.326 0.016	0.001 -0.276 0.016
		0.250 0.228 0.016	0.249 0.278 0.016
Panda #2	0.625 -0.025 0.016	0.476 -0.025 0.016	0.476 0.025 0.015
	0.626 0.025 0.016	0.476 0.150 0.015	0.476 0.200 0.016
		0.476 -0.200 0.016	0.476 -0.150 0.016
		0.626 0.150 0.016	0.626 0.200 0.016
		0.626 -0.225 0.016	0.626 -0.175 0.016
Panda #3	0.419 -0.027 0.020	0.522 -0.226 0.020	0.521 -0.176 0.020
	0.418 0.023 0.020	0.465 0.225 0.021	0.464 0.275 0.021
FR3	0.401 -0.029 0.017	0.399 -0.230 0.017	0.400 -0.180 0.017
	0.401 0.021 0.017	0.526 -0.030 0.018	0.527 0.020 0.018
Kuka iiwa 14	0.551 -0.026 0.009	0.437 -0.232 0.506	0.437 -0.232 0.556
	0.551 0.024 0.009	0.300 -0.452 0.009	0.300 -0.402 0.009
Kinova Genline3	0.030 -0.230 0.081	0.243 -0.071 0.206	0.239 -0.069 0.155
	-0.020 -0.228 0.082	0.254 0.084 -0.025	0.252 0.135 -0.022
		0.091 0.135 0.083	0.091 0.186 0.084

Table 1: Training and test socket positions for each of the calibrated robots in this study.

RC: Section IV.A states that the regularization optimization factor uses 10^{-4} but provides no rationale. There is no discussion of how lambda was tuned (e.g., via L-curve, grid search or cross-validation). Sensitivity analysis is absent: Do larger lambda values over-constrain the model (preserving nominal parameters at the cost of accuracy)? Do smaller values cause overfitting? Given that regularization directly impacts the optimization's balance between "minimal modifications of the nominal model" and calibration accuracy (Section III.C), this omission is critical for reproducibility.

AR: We thank the reviewer for highlighting the missing discussion on the importance and the choice of the regularization parameter. In the ablation study, we turned the regularization term on and off to investigate its effect and provide insight for future developments and the usability of the framework.

> *Moreover, we notice that the regularization acts as an extra safeguard layer that does not have a clear impact on the optimization in this case. To tune it, a good rule of thumb is to start from a very low value and only increase it if converging to a dimorphic URDF.*

We explained that the regularizer is necessary since we are running unconstrained optimization, and there is the risk of converging to a dimorphic URDF model that does not resemble the nominal model at all. The regularization value is kept very low during the experiments, since the models always converged to good models. However, the possibility of regularizing is available for future users of the algorithm and the framework.

RC: In the header of Table I (Section VI.A), the authors incorrectly refer to "Mean Absolute Error (MSE)". This is misleading: MSE (Mean Squared Error) and MAE (Mean Absolute Error) are distinct metrics. The formula in Section IV.A confirms the metric is MAE, not MSE. The acronym "MSE" is not defined on first use, violating standard academic practice.

AR: We thank the reviewer for highlighting the incongruence in the text. In the experiments section, we say:

> *Table 1 highlights how the mean absolute error (MAE) with respect to the socket position estimated as the mean of the model, i.e.*
>
> $$\frac{1}{N}\left(\sum_{i=1}^{N^0}\|\Phi_{\boldsymbol{\Theta}}(\boldsymbol{q}_i^0)-\boldsymbol{\mu}^0\|+\sum_{i=1}^{N^1}\|\Phi_{\boldsymbol{\Theta}}(\boldsymbol{q}_i^1)-\boldsymbol{\mu}^1\|\right)$$

However, in the methodology section, in order to formulate the variance-bias decomposition, we started from the mean square error definition. In the text, we say:

> *Given a vector of parameters $\boldsymbol{\Theta}$ and N^0 and N^1 measured joint configurations for the socket 0 and 1, the mean square error (MSE) with respect to the ground truth Cartesian position $\hat{\boldsymbol{X}}$ is*
>
> $$J_{mse}=\frac{1}{N}\sum_{i=1}^{N}\|\Phi_{\boldsymbol{\Theta}}(\boldsymbol{q}_i)-\hat{\boldsymbol{X}}_i\|_2^2 \quad (1)$$
>
> *where $N=N^0+N^1$.*

What we optimize is not directly the MSE but the available variance in each socket and the bias in the distance estimation between the two sockets.

RC: Section III.A ("Kinematics") introduces foundational equations for kinematic transformations but omits critical numerical identifiers. Key unnumbered formulas include "the transformation matrix for fixed joints", "The rotation matrix for revolute joints", etc..

AR: We thank the reviewer for the comment and the incongruence in the text. We added a number for any equation in the text.

RC: Section III.C ("Parameter Optimization") suffers from poorly defined symbols and inconsistent use of superscripts/subscripts.

AR: We thank the reviewer for highlighting the incongruence. We carefully reviewed the text and propose the updated version. Now all the equations are consistently using the same notation.

RC: In Section III.C, the cost function J combines terms like $tr(\Sigma_0)$, but the mean of tr() is not stated. Perhaps it denotes the trace of the matrix, however, some readers are not clear about the meaning of this symbol.

AR: We thank the reviewer for the comment. We updated the text specifying that it is true and why it is useful.

By using the variance-bias decomposition [2] of each of the two mean squared error costs, we obtain

$$J^0_{mse} = \underbrace{\mathrm{tr}(\boldsymbol{\Sigma}^0)}_{variance} + \underbrace{||\boldsymbol{\mu}^0 - \hat{\boldsymbol{X}}^0||_2^2}_{bias} \tag{2}$$

where $\mathrm{tr}()$ *is the trace operator. The trace of the variance matrix measures the expected squared radius of the cloud of points.*

Reviewer 2

RC: Section I, the authors emphasize that the proposed method " eliminating the limitation of relying on the average nominal model " but the specific limitations are not clearly defined.

AR: In the text, we now highlight that our approach is closely related to [1], still a completely new definition of the optimization paradigm. We are not optimizing the mean square error with respect to the average value of the original model for each socket. We highlight that the approach of [1] cannot fix the volumetric distortion of the original model.

The use of the average nominal model as ground truth is a strong limitation that retains volumetric miscalibration, hence, our proposed method gets rid of it.

RC: The experimental section should also include comparative experiments with this method to demonstrate the superiority of the proposed approach.

AR: To show this, we compare the optimization when using the mean square error versus the variance plus bias approach proposed in this article. We added a new box plot in the text, also reported here for simplicity in Figure 1. The reviewer would notice that the optimization in Petric et al. converges to a very low variance in the prediction but retains distortions in the model since it computes the target for the MSE using the (distorted) nominal model. On the other hand, the variance plus bias paradigm that we introduced fixes the issue.

RC: Equation (5) in Section III.C is the proposed cost function, consisting of inconsistency, distortion, and regularization terms. The first and second terms include the mean value μ. This appears to contradict the emphasis in Section I regarding eliminating reliance on the average. The authors need to further clarify how this effectively removes the limitation of dependence on the average.

AR: We agree with the reviewer on the missing comparison and clarifying what we mean by computing the mean in our cost function. The means in our cost functions are a function of optimizing parameters, and it is not computed with the original (inaccurate and distorted model).

When introducing the mean and the variance in the text, we say:

The mean vectors $\boldsymbol{\mu} \in \mathbb{R}^3$ and the variance matrices $\boldsymbol{\Sigma} \in \mathbb{R}^{3\times3}$ are a function of the optimization variable $\boldsymbol{\Theta}$ and are defined as

$$\boldsymbol{\mu}^{0,1} = \frac{1}{N^{0,1}} \sum_{i=1}^{N^{0,1}} \Phi_{\boldsymbol{\Theta}}(\boldsymbol{q}_i^{0,1})$$

and

$$\boldsymbol{\Sigma}^{0,1} = \mathbb{E}\big[\left(\Phi_{\boldsymbol{\Theta}}(\boldsymbol{q}^{0,1}) - \boldsymbol{\mu}^{0,1}\right) \left(\Phi_{\boldsymbol{\Theta}}(\boldsymbol{q}^{0,1}) - \boldsymbol{\mu}^{0,1}\right)^{\top} \big].$$

RC: In Section III.C, the authors state: " To minimize the volumetric distortion of the method, the only ground truth measure that is exploited in this calibration routine is the distance between the two sockets and the minimal movement of the ball's center point in a given socket." This highlights the critical importance of the MUKCa calibration tool's manufacturing precision and reliability. However, the device is solely 3D-printed. Concerns are raised regarding factors such as printing material, manufacturing accuracy, potential shifts in the ball center point during human operation, and tool

deformation under force. The authors should supplement relevant content to demonstrate the reliability of the MUKCa calibration tool.

AR: We agree with the reviewer. We highlight in the text that this has been possible thanks to incredible advances in the quality of 3D printing machines. The tolerances of modern, commercial 3D printers reach 0.1 mm accuracy. This allows for printing the socket and the sphere with minimal tolerance, and the distance between the two sockets is validated with a caliper (thanks to the printed notches) and has errors lower than 0.1 mm. For future works, it would be interesting to machine the components, add magnets for consistent robot positioning. This would not affect the contribution to formalizing the calibration procedure, as it would still involve only two sockets, no external measurement systems, and would not rely on the nominal model for target position estimation. However, in this paper, we aim to highlight how, even with a 3D-printed tool, incredible calibration performances can be achieved. This can democratize and make the tool accessible to anyone, without requiring the purchase of the tool. Everything proposed in this article is readily available for anyone to try.

RC: The parameter description and conclusion analysis in Section IV.A need for further enrichment and strengthening. It is suggested to supplement information such as the number of data points collected per socket and the distance between the sockets. For Figure 6, it is recommended to clearly specify the number of data points used for training or prediction at different workpiece positions and supplement relevant theoretical analysis.

AR: We thank the reviewer for highlighting the missing distance between the sockets.

> *We 3D printed the two sockets at distance of* $50\,\mathrm{mm}$ *and radius of* $20\,\mathrm{mm}$ *while the sphere of* $19.9\,\mathrm{mm}$. *The employed 3-D printer is a commercial entry-level BambuLab A1 set to the extra fine accuracy, i.e.,* $0.08\,\mathrm{mm}$.

Furthermore, we specify the number of joint configurations per socket:

> *The users are asked to use the computer keyboard to add an observation or the buttons on the robots (available only on Franka) with a minimum of thirty samples per socket.*

In the Appendix, we also highlight the number of recorded tool positions to test the position accuracy that are far away from the training position.

RC: In Section IV.B, the authors use a 10 mm diameter peg to perform peg-in-hole operations on holes ranging from 16 mm to 10.4 mm diameter to verify model consistency. The allowable clearance varied from 6 mm to 0.4 mm. While the experimental results effectively demonstrate the feasibility of the proposed method, its superiority requires comparative validation against state-of-the-art alternative methods.

AR: We thank the reviewer for highlighting this. We added a comparision with Petric et al [1]. The inability of the alternative method to calibrate the model distortion would render the experiment (in particular, when moving the peg to different locations on the table) ineffective.

RC: Section IV.B mentions: "The goal pose is computed by manually positioning the robot, with the elbow up, in a tight hole and recording the desired Cartesian pose from the forward kinematics." The authors first teach a robot configuration for peg insertion (where insertion error is approximately zero) and

then execute the peg-in-hole task 20 times at the same location with random configurations. Due to positional error similarity [3], it is suggested to supplement the distribution of robot configurations during the peg-in-hole task execution and use quantitative peg insertion error instead of the qualitative description "peg-in-hole success or failure." The drawing task description should undergo the same modification. This will enhance the persuasiveness and rigor of the paper.

AR: We thank the reviewer for the valuable feedback on the experiments. The insertion tasks and the repetitive motions are more qualitative demonstration on the impact of using a calibrated model. We however, use carefully printed peg and holes tools with measured tolerances. By measuring success and failure, we can claim that the robot error is lower than the radial difference between the peg and the hole. Moreover, we also moved the holes on a calibrated table. When asking to move the goal of the insertion of the know (calibrated) distance on the table, the insertion was still successful, highlighting that the model converged to a perfectly volumetric calibrated model. We added the suggested citation [3] to highlight that we did the insertion validation on not only in the proximity of the calibration position but also further away to that point.

We updated the text:

> *Moreover, to validate the volumetric calibration of the new model and that the robot is not only locally accurate [3], the hole was moved to 24 other positions positioned on an "X" shape (6 on each branch) on a calibrated table. The screws are at a distance of* $25\,\mathrm{mm}$ *in the x and y axes with an accuracy of* $0.1\,\mathrm{mm}$. *The x-y desired insertion coordinate is displaced according to the movement of the peg on the table. The experiment resulted in all successful 24 insertions, however, with a lower or higher interaction force due to the inaccuracy of the peg/hole devices, kinematic model, and calibrated table, with a mean force of* $6\,\mathrm{N}$ *and max force of* $30\,\mathrm{N}$ *and min of* $0.7\,\mathrm{N}$

RC: The method used in Section IV for robot accuracy verification differs from conventional large-workspace robot accuracy validation based on laser trackers. Please justify the rationality of the experimental verification approach.

AR: We agree with the reviewer. We did not use the laser tracker, as we wanted the validation of the model to rely solely on the MUKCa tool. That is why we test how well the model performs in consistently placing tools on the workspace. We agree with the reviewer that it would be valuable for future iterations to compare the parameter optimization obtained with MUKCa to that obtained with a laser tracker-based optimization.

Reviewer 3

RC: **While the method works very well for robots with Franka, KUKA, the performance degrades significantly on the Kinova robot. This limitation is acknowledged but could be better quantified and discussed in terms of applicability boundaries. The approach depends on several assumptions (backdrivable motors, negligible encoder noise, negligible socket distance uncertainty). These are reasonable for many cobots but may not hold universally. The sensitivity to violations of these assumptions should be more explicitly evaluated.**

AR: We thank the reviewer for highlighting the missing discussion. We would like to highlight that the calibration procedure also worked on the Kinova robot, reducing the original error. However, we do not reach the same sub-millimetric accuracy of franka and Kuka. The major reason is the joint backlash. We highlight in the assumptions that any unobservable joint movements are going to affect negatively the Cartesian estimation of the end-effector, hence also the calibration procedure. The violation of the assumption we provide usually affects the ability to calibrate the robot kinematics, but they are omitted in other state-of-the-art articles.

RC: **The paper notes that calibration accuracy degrades at positions far from the training location, especially with low-cost robots. While additional datasets improve performance, a more systematic analysis of workspace coverage requirements would be valuable.**

AR: We thank the reviewer for stressing this point. We tested at various points throughout the workspace for numerical evaluation. The supplementary material highlights all the socket positions that are used to test the accuracy prediction.

RC: **Please provide one figure in Section III for better understanding, as in [4].**

AR: We thank the reviewer, however, for a matter of space, we leave the illustration of the robot and the revolute parameters out, considering the reader can easily access the different URDF and geometry for different robots. However, we found the suggested publication to be very insightful and added it to the Method section when mentioning alternative formulations for forward kinematics, compared to using the URDF.

> *[...], or using Lie Theory [4], [...]*

References

[1] T. Petrič and L. Žlajpah, "Kinematic model calibration of a collaborative redundant robot using a closed kinematic chain," *Scientific Reports*, vol. 13, no. 1, p. 17804, 2023.

[2] C. M. Bishop and N. M. Nasrabadi, *Pattern Recognition and Machine Learning*. New York: Springer, 2006, ch. 4, pp. 147–152.

[3] Y. Zeng, W. Tian, and W. Liao, "Positional error similarity analysis for error compensation of industrial robots," *Robotics and Computer-Integrated Manufacturing*, vol. 42, pp. 113–120, 2016.

[4] Z. Fu, E. Spyrakos-Papastavridis, Y.-h. Lin, and J. S. Dai, "Analytical expressions of serial manipulator jacobians and their high-order derivatives based on lie theory," in *2020 IEEE International Conference on Robotics and Automation (ICRA)*. IEEE, 2020, pp. 7095–7100.

In this paper, the authors present a kinematic calibration approach (MUKCa) for collaborative robots, utilizing a 3D-printable two-socket tool and an optimization framework to enhance end-effector accuracy without relying on external measurement systems. The manuscript is well-structured, including a video presenting the calibrating environment and conditions, making it easy to comprehend and engaging for readers. The author has delineated the following three key contributions: (1) eliminating the limitation of relying on the average nominal model [10]; (2) avoiding the need for an expensive CMM to calibrate the distance between the sockets [8] or using an external measure to define the calibration tool frame; (3) removing the requirement for a costly auto-centering device for the spherical joint in the socket [9]. The manuscript is well-structured, including a video demonstrating the calibrating environment and conditions, making it easy to comprehend and engaging for readers.

However, while the concept of low-cost calibration is commendable, the work fails to demonstrate significant advantages over existing approaches, particularly the closely related reference [10] (Petrič et al.). The claimed contributions—eliminating the need for a third socket and avoiding reliance on nominal model to obtain the average positions of sockets — do not translate to measurable performance improvements. Inadequate presentation issues further undermine the paper's validity.

Some major recommendations:

* The manuscript reports that MUKCa reduces the mean absolute error (MAE) for Franka robots to 0.15–0.3 mm (Table I and Section IV.A). However, as shown in the last subfigure of Fig. 6, Petri's three-socket method [10] achieves comparable accuracy (0.2–0.3 mm) on similar Franka robots when trained simultaneously across all positions. This marginal difference (≤0.05 mm) is within typical experimental variability, offering no meaningful improvement in precision. The overlap in performance undermines the claim that MUKCa represents a significant advance. The authors emphasize the affordability and simplicity of a two-socket tool, but this advantage is negligible in the context of offline calibration. Both MUKCa and [10] require offline data collection (30+ samples per socket), and the incremental cost or complexity of adding a third socket (especially for 3D-printed tools) is minimal.

* In Section IV.A , the authors describe MUKCa uses random test positions "many datasets… in different parts of the workspace" without clear partitioning. As is well-

established in robot calibration, test data results will approximate training data performance when the test set closely resembles the training distribution. This principle necessitates spanning the entire workspace with training data during calibration. When comparing methods in Petrič[10], the authors explicitly evaluate calibration generalization by structuring training and testing datasets across defined workspace regions: "one in front of the table, one to the extreme left at table height, and one on the right." MUKCa does not adopt this protocol, instead using random cross-validation. This comparison is neither fair nor rigorous.

* In the abstract, the authors claim "… reduce the mean absolute error from the order of 10mm to 0.2mm for *both* Franka and Kuka robots." However, TABLE I shows that the result of mean absolute error for Kuka robot before calibration is 7.23×10^{-4} (training set) or 8.06×10^{-4} (test set). Although the authors do not specify the unit in TABLE I, based on the context, the unit in this table should be meters. The mean absolute error for Kuka robot, either Abstract or TABLE I, one of them must be incorrect.

* The variance-bias decomposition of mean squared error (MSE) in Section III.C is central to MUKCa's cost function but lacks supporting references. This decomposition is not usual to kinematic calibration, and readers require citations to foundational work (e.g., statistical learning literature on bias-variance tradeoffs) or an appendix with derivations to validate its application here.

* Section IV.A states that regularization optimization factor uses $\lambda = 1 \times 10^{-4}$ but provides no rationale. There is no discussion of how λ was tuned (e.g., via L-curve, grid search or cross-validation). Sensitivity analysis is absent: Do larger λ values over-constrain the model (preserving nominal parameters at the cost of accuracy)? Do smaller values cause overfitting? Given that regularization directly impacts the optimization's balance between "minimal modifications of the nominal model" and calibration accuracy (Section III.C), this omission is critical for reproducibility.

Some minor recommendations:

* In the header of Table I (Section VI.A), the authors incorrectly refer to "Mean Absolute Error (MSE)". This is misleading: MSE (Mean Squared Error) and MAE (Mean Absolute Error) are distinct metrics. The formula in Section IV.A

$$\frac{1}{N}\left(\sum_{i=1}^{N^{(0)}}\left\|\Phi_{\Theta}\left(q^{(0)}\right)-\mu^{(0)}\right\|+\sum_{i=1}^{N^{(1)}}\left\|\Phi_{\Theta}\left(q^{(1)}\right)-\mu^{(1)}\right\|\right)$$

confirms the metric is MAE, not MSE. The acronym "MSE" is not defined on first use, violating standard academic practice.

* Section III.A ("Kinematics") introduces foundational equations for kinematic transformations but omits critical numerical identifiers. Key unnumbered formulas include “the transformation matrix for fixed joints”, “The rotation matrix for revolute joints”, etc..

* Section III.C ("Parameter Optimization") suffers from poorly defined symbols and inconsistent use of superscripts/subscripts. For example, the definition for symbols X^i, $X^{(0)}$, X^0, and $X_i^{(0,1)}$ are unclear; $\Sigma_{(0)}$ and $\Sigma^{(0)}$ are the same symbol?

* In Section III.C, the cost function J combines terms like $tr(\Sigma_{(0)})$, the mean of tr is not stated. Perhaps it denotes the trace of the matrix, however, some readers are not clear about the meaning of this symbol.
